# Metal-Organic Frameworks-Based Sensors for Food Safety

**DOI:** 10.3390/foods11030382

**Published:** 2022-01-28

**Authors:** Aloys Hitabatuma, Peilong Wang, Xiaoou Su, Mengmeng Ma

**Affiliations:** Institute of Quality Standards and Testing Technology for Agro-Products, Chinese Academy of Agricultural Sciences, Beijing 100081, China; ahitabatuma@yahoo.fr (A.H.); wangpeilong@caas.cn (P.W.); 82101181092@caas.cn (M.M.)

**Keywords:** foodborne contaminants, food safety, food detection, metal-organic frameworks (MOFs), sensing

## Abstract

Food contains a variety of poisonous and harmful substances that have an impact on human health. Therefore, food safety is a worldwide public concern. Food detection approaches must ensure the safety of food at every step of the food supply chain by monitoring and evaluating all hazards from every single step of food production. Therefore, early detection and determination of trace-level contaminants in food are one of the most crucial measures for ensuring food safety and safeguarding consumers’ health. In recent years, various methods have been introduced for food safety analysis, including classical methods and biomolecules-based sensing methods. However, most of these methods are laboratory-dependent, time-consuming, costly, and require well-trained technicians. To overcome such problems, developing rapid, simple, accurate, low-cost, and portable food sensing techniques is essential. Metal-organic frameworks (MOFs), a type of porous materials that present high porosity, abundant functional groups, and tunable physical and chemical properties, demonstrates promise in large-number applications. In this regard, MOF-based sensing techniques provide a novel approach in rapid and efficient sensing of pathogenic bacteria, heavy metals, food illegal additives, toxins, persistent organic pollutants (POPs), veterinary drugs, and pesticide residues. This review focused on the rapid screening of MOF-based sensors for food safety analysis. Challenges and future perspectives of MOF-based sensors were discussed. MOF-based sensing techniques would be useful tools for food safety evaluation owing to their portability, affordability, reliability, sensibility, and stability. The present review focused on research published up to 7 years ago. We believe that this work will help readers understand the effects of food hazard exposure, the effects on humans, and the use of MOFs in the detection and sensing of food hazards.

## 1. Introduction

**Figure 1:**
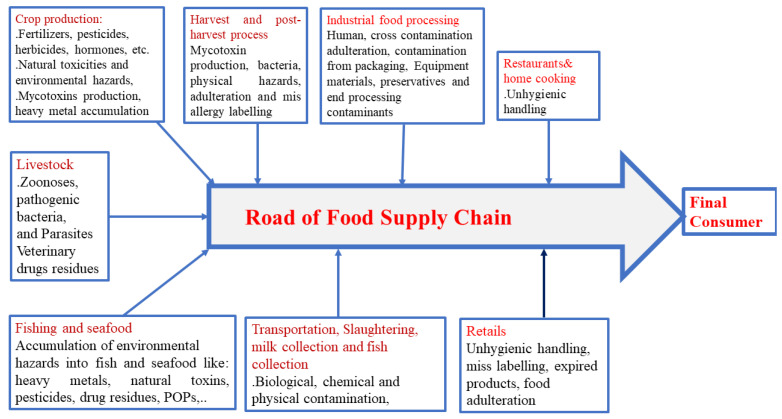
Prominent routes of food hazards and food contaminant exposure.

**Figure 2:**
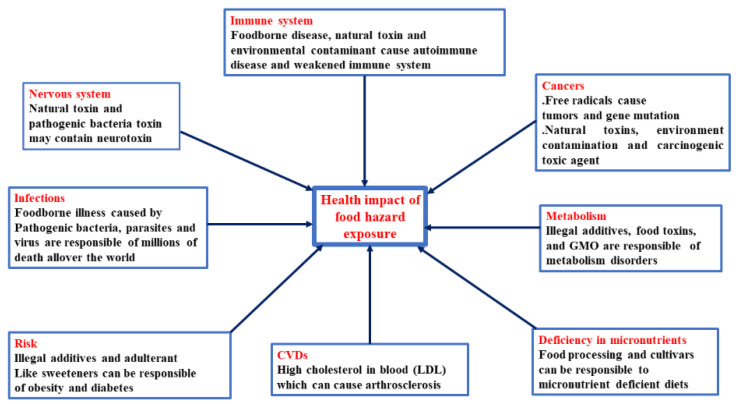
Health effects of food hazards and food contaminant exposure.

Food safety is currently one of the world’s most pressing concerns due to rapid urbanization and an increase in population. Such an over-increasing of population leads to the high demand for food production and commercialization, which also attracts significant attention to ensure food safety and quality control to meet consumer expectations toward decreasing the critical problem of foodborne disease [1,2]. According to the reports of World Health Organization (WHO), food regulations and safety measures have been taken into account of the global health concerns and the trend of foodborne disease has become a challenge that remains in outbreak investigation [1,3]. Major food hazards (biological, chemical, or physical) could enter into the food supply chain at any time during harvesting, processing, transporting, preparing, storing, and serving food (Figure 1). The main causes of foodborne disease are due to food hazards such as pathogens, heavy metals, toxic substances, POPs, pesticide, and veterinary drugs (Figure 2). Therefore, the detection and identification of hazardous substances in food are very important in inspections procedures and food control systems [4,5].

Thus, the best action by which to eliminate the foodborne disease is the early detection of food safety [6,7]. Consequently, several detection methods have been developed to control food quality and safety. The development of classical analytical methods such as high-performance liquid chromatography (HPLC), gas chromatography (GC), enzyme-linked immunosorbent assay (ELISA), lateral flow immunoassay, flow-through immunoassay, surface plasma resonance (SPR), and electrochemical immunosensors have shown positive contributions to the detection of food hazards [8,9]. All of these procedures, on the other hand, are laboratory-dependent, time-consuming, and require skilled workers, hence making these analytical methods inferior candidates for easy analysis. Therefore, the development of practical, cost-effective approaches for food hazards detection has a large impact on global food safety.

The utilization of chromogenic and luminescent Chemo sensors in food safety has partially met this demand [10,11]. Likewise, immune sensing, aptamer-based biosensing, and enzymatic techniques for food safety analysis have also been identified as effective sensing platforms [12,13,14]. The practicability of these detection approaches has been demonstrated with the recent development of advanced and new functional materials such as carbon nanotubes (CNTs), quantum dots (QDs), gold nanoparticles, graphene oxide (GO), and silver nanoparticles, etc. [15,16,17,18]. Such progress has contributed more in the development of efficient sensors for food safety analysis with fast response times and a wide range of applications in both liquid and solid phases [14,19,20]. However, these advanced sensors also present several drawbacks, such as a complicated synthesis procedure, poor photostability, deficiencies in molecular organization, and frequent interference from other analytes.

Recently, nanotechnologists have reported different innovated types of sophisticated materials for specific applications. Among them, metal-organic frameworks (MOFs) have emerged as cutting-edge materials for analytical sensing. MOFs are a class of crystalline porous materials systems structured by using metals linked together by organic bridging ligands, which have received considerable attention in the past years. They have proven successful in various applications, such as gas storage and gas separation, catalysis, energy storage, contaminant removal, chemical sensing, drug delivery, and bioimaging [21,22,23]. MOFs represent a niche in the nanomaterials field due to their properties, which allow them to be specifically tailed, which is extremely valuable in the field of food safety analysis. MOFs have demonstrated their versatility and have been developed for use in the detection and monitoring of contaminants in food. As discussed, and reported by the previous researchers, MOFs present uniform and stable structure, high selectivity, tunable porosity, and luminescent characteristics, which qualify them to be used as advanced sensing materials for food safety analysis compared with other nanomaterials [24,25,26,27], e.g., carbon nanotubes (CNTs), quantum dots (QDs), gold nanoparticles, graphene oxide (GO), and silver nanoparticles. MOF-based sensors have huge capacities for post-synthetic modification, possible activation of pendant groups, suitable signal transduction, and capacity of biofunctionalization [28]. Moreover, the availability of the functional groups after biofunctionalization in frameworks coupled with the luminescent is practicable exploitation and is a very attractive field of research [29].

Recently, authors have reviewed applications of MOFs in food safety monitoring from sample preparation, separation, packaging, and storage to detection and cleaning [24,25,27]. We intend to present the most recent advances and challenges in the use of MOFs as potential sensing materials for food safety analysis. In this review, the sources of food contamination and health effects of food toxicology were discussed; the sensing principles of MOFs were described along with areas of practical applications in food safety analysis. Specific attention was paid to the MOF-based sensing methods, developed for the detection of pathogenic bacteria, heavy metals, adulteration, toxins, drugs, and pesticide residue and persistent organic pollutants (POPs). Besides, the essential attributes of MOF-based sensors for high value-added applications in different fields were discussed and new perspectives for decreasing the risk of foodborne illness were highlighted.

## 2. Food Exposition to Hazard and Food Contaminants

Pollution in the environment caused by various hazards poses a serious health risk and endangers public health and food safety (Figure 1). Inadequate detection of food contaminants may have a negative impact on public health and economic development in various countries. Pathogenic bacteria, heavy metals, illegal food additives, toxins, veterinary drugs, POPs, and pesticide residue are the most commonly reported food hazards today (Table 1). Currently, one of the most serious issues affecting public health and food safety is the outbreak of foodborne diseases caused by the consumption of contaminated food containing food hazards [30].

According to the estimation of a WHO report, contaminated food containing pathogenic bacteria, viruses, parasites, or chemical substances are potentially inciting agents of 200 diseases varied from diarrhea to cancers. Its estimated that 600 million (around 1 of 10 people in the world) fall sick after consumption of unsafe food [31]. Foodborne disease, food illness, or food poisoning refers to any illness caused by biological hazards (pathogenic bacteria, viruses, and parasites) or chemical hazards (heavy metals, natural toxins, veterinary drugs, pesticide residue, and food adulteration) that contaminate food (Figure 2). Food-borne disease is primarily caused by improper food handling, preparation, or storage [32].

**Table 1:** Occurrence and health effects of food hazards and food contaminants.

Food Hazards	Harmful Health Effect	Source of Contamination	Most Contaminated Food	Control Majors	Reference
**pathogenic bacteria**					
*Salmonella enterica* serovars Thphimurium	Food toxin and typhoid fever	Fecal contamination, eating raw, or inadequately cooked food or contaminated water	dairy product, meat, eggs, vegetables and processed food, and untreated water	Frequent handwashing, consuming treated water, and well-cooked food served hot	[33]
*Salmonella enterica* serovars	Gastroenteritis and bloody diarrhea	Eating raw or inadequately cooked seafood or other contaminated food and water	Seafood and water	Eating cooked seafood and other foods and drinking treated water	[34]
*Shigella dysenteriae*	Epidemic bacillary dysentery and Shigellosis	Inadequate water and poor sanitation	Water and contaminated food	Frequent handwashing, drinking and using treated water	[35]
*Escherichia coli* O157:H7	Produce shiga toxin which can damage lining of intestine	Contaminated water and raw food	Meat products, dairy products, juice, fruits and vegetables	Consuming well-cooked food served hot	[36]
*Listeria monocytogenes*	Listeriosis	Raw food and having the ability to resist low temperatures	Meat and meat products, dairy product, fruits and vegetables	Consuming cooked food and treated milk	[37]
*Shigella sonnei*	Shigellosis, bacteria dysentery, diarrhea, tenesmus, and toxic shock	Fecal contamination caused by unproper hygiene	Fresh fruit and vegetables, raw oysters, deli meats and unpasteurized milk	Good hygiene practice during food handling	[38]
*Staphylococcus aureus*	Food poisoning, skin infection, Animal infection, Bacteremia and Bone and joint infection	Close contamination caused by unproper hygiene	Milk and dairy products	Consuming pasteurized milk and milk products	[1]
*campylobacter jejuni*	Bacterial gastroenteritis, autoimmune neurological disorders like Guillain-Barre syndrome, Miller Fisher	Consumption of undercooked meat and meat products and other contaminated food	Meat products, especially poultry products	Consuming cooked meat	[39]
**Name of Heavy Metal**					
Pb^2+^	Interfering with proper enzymes function, anemia, insomnia, irritability, memory loss, weight loss, hearing loss, loss of coordination, etc.	Environmental and water pollution	Water, beverages, fruits and vegetables, cereal products	Using and consuming tested water and food	[40]
Hg^2+^	Neurotoxin, acrodynia, Hunter-Rusell syndrome, damaged brain, kidney, and lungs	Environmental and water pollution	Water, beverages, fruits and Vegetables, cereal products	Using and consuming tested water and food	[41]
K^+^	Abnormal concentration causing kidney disease, heart disease, diabetes, anorexia, bulimia, blood high pressure, stroke, Addison’s and adrenaline gland disease	Environmental and water pollution	Water, beverages, fruits and vegetables, cereal products	Using and consuming tested water and food	[42]
As^3+^	Causes cancer of the skin, lung, urinary bladder, liver, and kidney	Environmental and water pollution	Water, beverages, fruits and vegetables, cereal products	Using and consuming tested water and food	[43]
Cd^2+^	Metal fume fever, pneumonitis and pulmonary edema	Environmental and water pollution	Cereal products, water, beverages, vegetables and fruits	Using and consuming tested water and food	[44]
**Natural Toxin**					
*Staphylococcus aureus* enterotoxin A	Gastrointestinal, severe allergic, auto immune response and toxic shock syndrome	Produced by Staphylococcus aureus	Milk and dairy products	Consume pasteurized milk and milk products	[45]
T-2 Toxin	Emesis, diarrhea, necrosis, cartilage damage, immunosuppression and apoptosis	Secondary metabolite of fusarium	Barley, wheat, maize, oats	Consumption of tested cereals product	[46]
Aflatoxin B1 (AFB1)	Cirrhosis, necrosis and carcinoma of liver	Secondary metabolites of *Aspergillus flavus* and *Aspergillus parasiticus*	Fruits, cereals, wine nuts, spices and soy products	Consumption of tested cereals food	[47]
Ochratoxin (OTA)	carcinogenic, hepatotoxic, teratogenic, nephrotoxic and immunotoxin	Secondary metabolites of *Aspergillus ochraceus*, penicillium verrucosum and penicillium nordicum	Wheat, corn, beans, wine, cereals and cereals products milk and milk products meat and meat products	Consumption of tested cereals food	[48]
Fumonisin B1	Carcinogen to human, leukoencephalomalaciia to horses and pulmonary edema to swine	Produced by more than ten species of Fusarium. *F. verticillioides* and *F. proliferatum* produce high concentration	Cereals and cereals products soybean and soy product	Consumption of and feeding tested cereals food	[49]
Okadaic acid (OA)	Immunotoxic and tumor promotion, diarrhea	Produced by harmful algal blooms (HABs)	Seafood	test seafood before consumption	[50]
Tetrodotoxin (TTX)	Neurotoxin and carcinogenic toxin	Produced by harmful algal blooms (HABs)	Seafood and water	test seafood and water before consumption	[51]
Microcystin-LR (MC-LR)	Cause live damage	Produced by cy	Seafood and water	test seafood water before consumption	[52]
β-lactoglobulin	Allergen	Milk allergen	Milk and milk products	Test and food labeling	[53]
Ricin toxin	Deadly plant toxin via inhibition of protein synthesizes, ribosome inactivation, dysphagia, hematemesis, and hypovolemia	Produced by castor beans (*Ricinus communis*)	castor beans	Food testing	[54]
Abrin toxin	Deadly plant toxins through ribosome and proteins inactivation,	Produced by peas (*Abrus precatorius*)	Rosary peas (*Abrus precatorius*)	Food testing	[55]
Botulinum toxins	Paralysis, arrhythmia, heart attack and respiratory arrest	Nerve toxin produced by the bacterium clostridium (*c. botulinum*)	Dairy products, vegetables, fruits, seafood, canned foods	Consume cooked and treated foods	[56]
Dopamine	Severe Psychiatric disorder, depression, schizophrenia and euphoria		Milk and milk products, meat and meat product	Early testing	[57]
Staphylococcus aureus enterotoxin C1	Diarrhea, vomiting and abdominal pain	Produced by *S. aureus*	Milk and milk products, meat and meat product, fruits and vegetable	Early testing and good hygiene practice during food handling	[58]
**Food adulteration**					
Melamine	Kidney failure	Food adulteration	Milk and milk products, meat, and meat products	Early testing	[59]
**Veterinary drugs and pesticides residues**					
Kanamycin	Ototoxicity, nephrotoxicity, allergic reaction to the drugs, vomiting, diarrhea, blurring of vision, and malabsorption syndrome	Animal breeding are stable resistance to decomposition, and elimination from biological systems	Meat and meat products and dairy products and eggs	Usage of appropriate dose and food testing	[60]
Chloramphenicol	Aplastic anemia and bone marrow suppression	Veterinary antibiotic used in animal breeding	Meat and meat products and dairy products and eggs	Usage of appropriate dose and food testing	[61]
Ractopamine	Muscle tremors, tachycardia, headache, cardiovascular and nervous system	Feed additives which are stably resistant to decomposition and elimination from biological systems	Meat and meat products and dairy products and eggs	Usage of appropriate dose and food testing	[62]
Streptomycin (Str)	Nephrotoxicity, Ototoxicity, vomiting and rash	Veterinary medicine used in animal breeding	Meat and meat products and dairy products and eggs	Usage of appropriate dose and food testing	[63]
Tetracycline	Allergen, bacteria drugs resistance	Veterinary antibiotic used in animal breeding	Meat and meat products and dairy products and eggsMeat and meat products and dairy products and eggs	Usage of appropriate dose and food testing	[64]
Organophosphorus pesticides	Tumors, genital change, blood and nerve disorders, endocrine disruption, coma, and leukemia	Used in agricultural pest control	cereal products, beans, coffee, fruits and vegetables	Limitation of its utilization and food testing	[65]
Acetamiprid	Carcinogenic, mutagenic and neurotoxic	Used in agricultural pest control	cereal products, beans, coffee, fruits and vegetables	Limitation of its utilization and food testing	[66]
Malathion	Carcinogenic	Used in agricultural pest control and mosquito control	cereal products, beans, coffee, fruits and vegetables	Limitation of its utilization and food testing	[67]

### 2.1. Pathogenic Bacteria

Pathogenic bacteria are the main source of foodborne disease. *Campylobacter jejuni*, *Clostridium perfringens*, *Salmonella* spp., and *Escherichia Coli 0157:H7* are the most common causes of foodborne disease. *Bacillus cereus*, *Escherichia coli*, *Listeria monocytogenes*, *Shigella* spp., *Staphylococcus aureus*, *Staphylococcal enteritis*, *Streptococcus*, *Vibrio cholerae*, *Vibrio parahaemolyticus*, *Vibrio vulnificus*, *Yersinia enterocolitica*, *Yersinia pseudotuberculosis*, *Brucella* spp., *Coxiella burnetii*, and *plesiomonas shigelloides* are other groups of pathogenic bacteria that are responsible for different foodborne diseases globally [31]. Moreover, some foodborne diseases do not directly originate from direct bacterial infection, but enterotoxins which target the intestines. Enterotoxins can cause illness even when the bacteria that produced them have been killed. The appearance of symptoms depends on the toxin but can be rapid at the onset, as in the example of enterotoxins of Staphylococcus aureus where symptoms can be observed in one to six hours [31,68]. *Clostridium botulinum*, *Clostridium perfringens,* and *Bacillus cereus* appear rarely but cause potentially deadly disease. Botulism presents when anaerobic, pathogenic bacteria *Clostridium botulinum* nurtures in inadequately canned foods with low-acid content and produces a powerful paralytic toxin called botulin [31].

These pathogenic bacteria are found in nature, including the environment, workplace, materials, and even on our clothes and hands. Furthermore, as various scientists have demonstrated, pathogenic bacteria can survive and persist in dry food and cause foodborne diseases [69,70]. Furthermore, antibiotic-resistant bacteria and climate change pose additional challenges to food safety. It has been discovered that improper antibiotic use can result in the development of antibiotic-resistant bacteria. Misuse and overuse of antibiotics in animal husbandry, in particular, could be a major cause of antibiotic resistance in the food chain [70,71]. Various research has been conducted on the impact of climate on foodborne diseases, especially on its influence on the development and gene mutation of pathogenic bacteria [72,73]. Climate change may impact the flowing elements epidemiologic triad: climate change can affect development and resistance of pathogenic bacteria in environments and the bacterial ecology and food matrix, etc. [72,73,74,75]. Thus, early screening and characterizing of pathogenic bacteria are necessary for clinical diagnosis, environmental monitoring, and food safety analysis.

### 2.2. Heavy Metals

Heavy metals are elements distributed in trace quantity in nature, and some of them in small concentrations play an important role for humans, yet they can cause toxicity when exceeding the recommended level. Among heavy metals Cu, Fe, and Mn are important for human life; these elements are coenzymes and natural, essential substances for growth and respiration. Contrarily, Pb and Cd considered as very toxic food contaminants and have biological importance and are sources of serious adverse health effects in humans [76,77]. It has been reported that Pb can be accumulate in erythrocytes and replace Zn as one of the important enzymes in heme biosynthesis called δ-aminolaevulinic acid dehydratase [78,79]. Additionally, researchers reported that Cd can induce carcinogenic diseases like pancreatic cancer and thyroid cancer [80]. Heavy metals can be taken into the living organism by ingestion, inhalation, and dermal absorption, which then might cause toxicity when exceeding the recommended limit. However, food and water are the primary sources of heavy metals exposure to the human body [81,82]. Heavy metals in the environment (soil and water) have increased significantly as a result of biomagnification and accumulation into foods as a result of anthropogenic, geological, and industrial activities. Heavy metals were recently identified as one of the most common contaminants found in packaged food and beverages, according to studies [83]. The main sources of foods’ contamination by heavy metals include the content of heavy metals in used unprocessed food (raw materials), which may result in the contamination of agricultural soil and irrigation water [84,85], and the extreme use of pesticides and fertilizers during crop production as well as feeds consumed by animals [86,87]. Additionally, contaminated water is used during food processing, packaging materials, in food-contact materials used in processing, and in used processing technologies (Table 1) [86,88]. Therefore, to maintain consumer safeguards, the levels of heavy metals should be regularly and rapidly monitored in many food materials.

### 2.3. Illegal Food Additives

Illegal food additives or adulteration in food has been a major concern in the food industry since the dawn of civilization, as it not only degrades the quality of food products but also poses a significant burden on public health and the global economy. Food adulteration is defined as the addition or removal of any substance to/from food that affects the natural quality and composition of the food substance [89,90]. According to the Food Safety and Standards Act (FSSA) of 2006, food is adulterated when there is evidence of substandard quality, substitution with a cheaper substance, abstraction of any constituent article, preparation or storage in unsanitary conditions, presence of poisonous ingredients, use of coloring agents and/or preservatives in excess of prescribed limits, or when quantity or purity is less than the prescribed standards. Food adulteration not only defrauds the consumer, but it is also a serious source of health risks that can lead to death [91,92].

Recently, various food adulteration or food contamination scandals have been reported and have occurred in many countries around the world. For instance, China has experienced various scandals including the recovery of gutter oil in 2011 and the adulteration of melamine into milk powder in 2008, which affected 300,000 babies, causing 51,900 hospitalizations and six infant deaths. Europe experienced a scandal involving adulteration of beef with horse meat in 2013, and various cases of olive oil fraud have also occurred in many countries. Besides, mass media have played a big role in reporting and advising the consumer on different food adulteration cases encountered around the world. Therefore, scientists analyzed and reported various food scandal events. Zhang and Xue (2016) conducted an aggregated analysis on economically motivated food frauds and adulteration in China by using 1553 media reports on food safety scandals and concerns. By using a systematic approach, the country’s food adulteration and food frauds reported cases were analyzed. Results indicated that economically motivated food fraud and adulteration is an emerging and serious food safety problem in China [93]. Additionally, Peng et al. (2016) reviewed scandals of major food adulteration in Taiwan between 2011 and 2015 and among them, food adulterations involving illegal additives were the most frequent [94]. Therefore, the development of convenient, cost-effective, food illegal additives detection methods has an impact on global food safety and the economy.

### 2.4. Mycotoxins in Food

Foodborne intoxications are one of the most prevalent risks to public health and have been increasing worldwide [95,96]. Natural toxins are a diverse group of molecules produced by fungi, plants, or microbiology that are toxic to humans or other vertebrates. Some of these molecules’ poisonous effects can be severe even at very low doses. Mycotoxins, a chemically heterogeneous group of fungal origin, are the most important class of natural toxins. Mycotoxin is thought to contaminate approximately 25% of crops [97]. Molds are not foodborne pathogens by themselves, but they can produce an array of secondary metabolites (mycotoxins) with acute or chronic toxicological effects. Fungi are a large group of diverse eukaryotic organisms which include yeasts and molds. Molds (filamentous fungi) are widely distributed in nature. Due to their versatile nutritional requirements, they are common contaminants and, under favorable conditions of humidity and temperature, propagate on different food commodities and beverages and produce mycotoxins. Molds can be easily grown on food products like cereals, coffee, beans, nuts, vegetables, and fruits [98,99].

Mycotoxins are a group of naturally occurring toxic compounds produced by the secondary metabolism of many filamentous fungi (mainly produced by six genera, including the *Penicillium*, *Fusarium* and *Aspergillus* genera) [96,100]. Both fungal growth and mycotoxin production depend on a variety of factors. The molds produce various types of mycotoxins such as aflatoxins (AFs), deoxynivalenol (DON), zearalenone (ZEA), fumonisins (FBs), ochratoxin A (OTA), and citrinin (CIT), with almost all being toxic to humans. It has also been reported that one type of mold may produce different types of mycotoxin and with its production being affected by various factors.

Mycotoxin contamination occurs throughout the entire food chain, from processing to transportation and storage [101]. Mycotoxin contamination is a worldwide problem in terms of human and animal health, as well as a significant economic burden on industry. Mycotoxins can contaminate a product throughout the food chain, both in the field and during storage, or at a later stage [102]. Contamination of human food and livestock feed by fungi and their toxins is a serious food safety issue worldwide. This results in enormous yield and economic losses, as well as acute or chronic toxicological effects [103]. Mycotoxins can contaminate crops prior to harvest or during post-harvest storage, and their consumption can cause acute or chronic toxicological effects in a variety of species, including humans, poultry, swine, and fish, resulting in varying levels of mortality and morbidity [104]. Developing effective sensors and detection methods for monitoring food mycotoxins contamination is therefore highly necessary.

### 2.5. Drug and Pesticide Residues

A pesticide is any substance or organism (including organisms derived from biotechnology) used to control, destroy, repel, or attract a pest or to mitigate the effects of a pest. A pest is defined as a plant, animal, or other organism that is either directly or indirectly harmful, noxious, or bothersome [105]. Insecticides, fungicides, and herbicides are examples of pesticides. One major issue with pesticides is that they can accumulate in the food chain and contaminate the environment. A veterinary drug is defined by the Codex Alimentarius Commission as any substance applied or administered to any food-producing animal, such as meat or milk of animals, poultry, fish, or bees, whether for therapeutic, prophylactic, or diagnostic purposes, or to modify physiological functions or behavior [106].

Veterinary drugs are typically used in food-producing animals to control and or prevent illness in the animals. An antibiotic can be defined as a chemical compound that kills or slows down the growth of a microorganism, and antibiotics are widely used in the treatment of various bacterial infections. The digestion of these drug residues or their metabolites could be considered harmful to humans [107]. Residual levels might not cause direct adverse health effects if ingested by consumers over their lifetime. The detection of these residues at trace amounts is the most important task in their monitoring and evaluation.

### 2.6. Persistent Organic Pollutants (POPs)

Persistent organic pollutants (POPs) are organic compounds with high toxicity to animals and humans that are persistent in nature because they are not biodegradable (resist photolytic, chemical, and biological degradation) in nature. As a result, they remain in the environment for longer periods of time, even at very low concentration levels. When POPs are exposed, they move up the food chain from lower trophic levels to higher trophic levels and biomagnify. POPs are naturally lipophilic, which allows them to bioaccumulate in organisms’ fat tissues. Furthermore, POPs can be easily transported long distances over regions far removed from their original production site via mediums such as air and water, making them a regional, national, and global concern [108,109].

Organochlorine pesticides (OCPs), polycyclic aromatic hydrocarbons (PAHs), polychlorinated biphenyls (PCBs), brominated flame retards (BFRs), dioxins, and dibenzofurans are the head pollutants that cause persistent organic pollutant contamination [110]. Most of these compounds have been used in industry and agriculture for a long time and are widely distributed in nature. Once they enter into food chain they will be accumulated in the fatty tissue of the human body and pose a risk to cause adverse effects to human health [111,112]. As a result, POPs are a body burden, particularly those used as insecticides or fungicides, which can be attributed to our primary dietary exposure. It has been reported that POP exposure is linked to a wide range of negative human health effects, including increased mortality [113,114], increased risk for type 2 and gestational diabetes, hypertension, and obesity [110,115]. According to an observational study carried out by analyzing results from a WHO-coordinated survey on POPs in human milk in Belgium, maternal age and BMI were usually associated with higher POPs concentrations. In addition, the POPs concentration in human milk corresponded with the level of POPs in the consumed diet [116]. Therefore, the development of novel and faster response methods with high sensitivity and selectivity that can be used in food and environment is crucial.

## 3. MOF-Based Sensors for Food Safety

**Figure 3:**
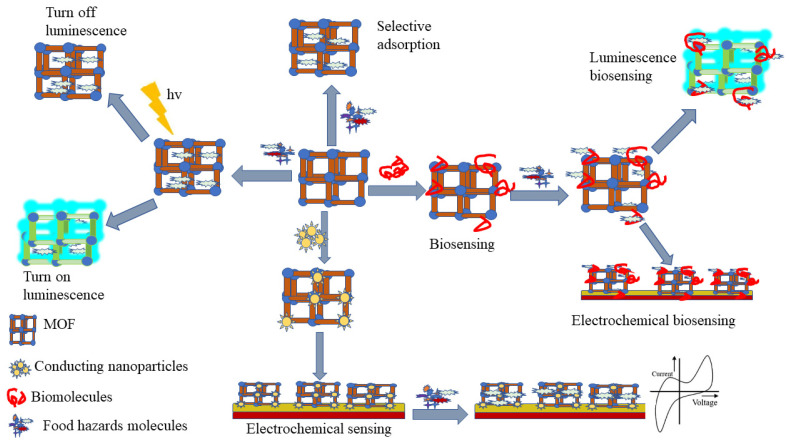
Schematic representation of various MOF-based techniques used for food safety analysis.

Over the past decades, there have been many studies and reports on MOF synthesis for food safety analysis (Figure 3), encompassing MOF designing, various synthesis methods, and post-synthetic modification, as well as incorporation of biomolecules into MOF [23,117]. Isoreticular expansion, topology-guided design, and modulated synthesis are the most reported methods for MOF synthesis. While, four main classes of post-synthetic modification include covalent post-synthetic modification, post-synthetic metalation modification, dative post-synthetic modification, post-synthetic exchange, and post-synthetic deprotection, and have been reported as tools to overcome different barriers for the application of MOF in food safety analysis [28,118,119]. Besides, incorporation of biomolecules into MOFs has been used as a new strategy to improve MOF efficiency in selectivity, sensitivity, signal amplification, and MOF stability [117]. To accommodate the drawbacks of the lower framework stability of MOFs, carboxylate-based linkers and N-heterocyclic based linkers have been used [120,121].

### 3.1. MOF-Based Electrochemical-Sensing Method

The Electrochemical sensing method is one of the major areas in the analytical method, since it easy, reliable, and cheap compared with other analytical methods. Therefore, recent years have seen a great rising of a scientific reports in the development and application of electrochemical-sensing techniques for the successful detection of food safety contaminants [122]. The selectivity and sensitivity of electrochemical methods prove them to be the best candidate for efficient food safety analysis. However, the effectiveness of the electrochemical sensing method is built based on the electrochemical properties of the transducer MOFs or nanomaterials (NMs) (redox reactions of the analyte in electrochemical system). Additionally, the conductivity properties of NMs govern the sensitivity of the electrochemical sensors [123,124]. Therefore, a great effort has concentrated on the improvement of MOFs’ conductivity properties to facilitate the design and synthesis of better and more sensitive MOF-based electrochemical sensors [125,126].

The research on MOF-based methods and electrical conductivity is still in its early stages. As a result of the lack of electrical conduction in their pristine forms, a large number of pre-existing MOF-based sensors are suitable for optical transduction. The organic linkers are redox-inactive and are attached to the hard-metallic cluster via hard oxygen-containing groups. As a result of their insulating properties, pristine MOFs are poor electrical conductors [127,128,129]. To overcome such challenges, various strategies and modifications to increase electrical conductivity have been introduced, such as doping MOFs with specific materials such as nanotubes, nanoparticles (NPs), and selected ionic species [130] (Table 2). The large specific surface area of the MOFs substrate makes it easier to load nanoparticles, which helps to improve conductivity and amplify electrical signals. For incidence, Talian et al. (2014) reported a realizing tunable electrical conductivity strategy in MOFs, where tetracyanoquinodimethane were used as organic linkers and tetrathiafulvalene were also used as organic linker by Narayan (2102) [129,131]. These changes can be used to improve the conducting properties of MOFs in order to develop potential electrochemical sensor technology based on MOFs [132]. MOF-based sensors can thus be post-modified by modifying their conductive properties and good absorption properties [132].

### 3.2. MOF-Based Chemical Sensing Method

A chemical sensor method is self-sufficient to provide chemical information of its environment through analytical reaction, whether it is the liquid or the gas phase of the surrounding environment [158]. Luminescent chemical sensing using MOFs has been reported as potential chemical sensors due to their easily induced luminescence, various advantages in structural and components, and their detecting mechanism [23]. Metal ions, organic ligands, and guest species (luminescent guest molecules or nanoparticles) are the most common source of MOFs’ luminescence. Light-emissive organic ligands containing aromatic or conjugated moieties as the linker and lanthanide ions are the most commonly used to fabricate luminescent MOFs [158]. Given that luminescent MOFs (LMOFs) detection capability can be enhanced by host-guest interactions, they have been proposed as excellent candidates for food safety analysis applications. In recent years, prospective applications of LMOFs have been investigated. The sensitivity of MOF-based detection of food contaminants is determined by the sensing method used for signal transduction [159].

Naturally, the sensitivity of LMOFs is linked to MOFs’ high loading capacities and analyte transport facilitation within their structural framework. Furthermore, active analyte incorporation into the MOF framework affects the limits of detection (LODs) for LMOFs [22,126]. Scientists have demonstrated that the fundamental mechanisms involved in the LMOF-based sensing approach are based on variations in the intermolecular distances between the metallic centers and the organic linkers, chemical interactions between the target analyte and the metallic clusters in the MOF framework, and host-guest interactions between the organic ligands and the guest analyte. The luminescent MOF’s working mechanism was mainly based on the occurrence of the fluorescence quenching method. Forster resonance energy transfer (FRET), photoinduced electron transfer (PET), inner filter effect (IFE), and competition of excitation light between MOF and analyte are the most popular quenching mechanisms for fluorescence quenching of MOF-based sensors [27,126,160]. All of these mechanisms are visible through a variety of luminescence-related phenomena such as ligand-localized emissions, ligand-to-metal charge transfer, metal-to-ligand charge transfer, plasma-induced gate oxide damage, sensitization, and metal/excimer/exciplex emissions [158,161].

Furthermore, LMOFs have one intriguing structural component: organic ligands are small, and these ligand molecules can self-quench, resulting in a higher electro-photoluminescence (PL) quantum yield [158]. A subset of LMOFs have a MOF structure that includes a stabilizing organic ligand with a tuned highest occupied molecular orbital and lowest unoccupied molecular orbital energy gap, resulting in a PL quantum yield value closer to one [158,162]. Furthermore, the use of organic linkers in MOFs that can absorb ultraviolet (UV)/visible light can result in fluorescence. Turning off fluorescence is the most common optical intensity quenching method used for signal transduction of LMOFs [162]. This quenching ability is thought to be caused by the overlapping of acceptor and donor electrons. Charges in the redox potential of the in-build moieties, on the other hand, have been recognized to account for quenching in some cases [163]. Furthermore, but not always, the luminescence intensity of the LMOFs increases in turn on fluorescence upon interaction with the guest analyte. This property can be used to quantify the target concentration at the same wavelength as the luminescence intensity increases [163,164].

The interaction MOF-analyte is accompanied by changes in physicochemical properties such as optical and electrical conductivity. Furthermore, LMOF-based optical detection can generate detection signals that can be seen with the naked eye. Overall, the ability to control the charges in the optical characteristics enables high sensitivity detection with low LODs, and this option has been adopted for food safety analysis (Table 3) [164,165]. Nonetheless, LMOF-based food safety analysis warrants future research and development due to identified drawbacks such as variation in the quenching rate and pathways along with medium stabilization and detrimental porosity [165,166,167].

### 3.3. MOF-Based Biosensing Method

The incorporation of biomolecules into sensing technology has resulted in biosensors being a cost-effective and time-efficient technique for food safety analysis. A biosensor is a self-contained, unified device that contains all of the subsystems required for electronic quantification and data transmission. Interactions of biomolecules that act as biological recognition elements and electrochemical transducers can produce a usable signal [183,184]. MOFs, porous crystalline materials built from the coordination of organic ligands and inorganic metal ions or metal, present ordered and tunable porosity, good crystallinity, and high surface areas, making them excellent for host matrix immobilization of biomolecules [117,185,186,187]. These excellent and unique MOFs’ properties give them outstanding support ability to incorporate biomolecules for modern food safety detection. Some food contaminant molecules can inhibit the activity of specific enzymes use to quantify a targeted analyte. The biosensing approach for food contamination sensing utilizes various kinds of biomolecules and therefore they have been successfully incorporated with MOFs [188,189,190,191,192,193]. Thus, the MOF-based biosensing approach for food safety analysis usually utilizes various biomolecules such as enzymes [194], antibodies (ab) [189,190], peptides [195], bacteriophages [196], and aptamers [11] (Table 4).

MOFs must be conjugated to biorecognition elements before they can be used in the development of biosensors. These biofunctionalizations can be achieved by using pendant functional groups from linkers’ moiety of MOFs. This procedure, however, provides unnecessary control over the functionalization reaction and may result in a bulk functionalization reaction rather than the intended surface modification. Scientists recently demonstrated that coating the surface of MOFs with silica can improve the condition of their biofunctionalization [211,212,213]. For incidence, the silica coating can play a double role: the improvement of the water stability and dispersibility of the MOFs and the facilitation of their effective surface functionalization. Based on these advantages, the thin assembly of silica-coated water-stable CU_3_(BTC)_2_@SiO_2_ on a conducting substrate was firstly reported [190,211]. Immunosensing has opened up new ways for MOF-based biosensors, in which antibodies serve as identification receptors. They are, of course, organic compounds that regulate peripheral physicochemical properties and govern the grafting procedure in order to improve the sensitivity and selectivity of the biosensing approach [189]. Recently, the development of impedimetric immunosensors technics has been exciting research field due to its ability to prove lab-on-chip devices that are not only easy to be integrated with microfluidic sample chambers, but also easy to calibrate [213,214]. Interestingly, MOFs can be used as nanosized electrode materials in the impedimetric immunosensor fabrication based on their hierarchical chemical assembly and availability of functional groups on them [213].

Aptamer-based sensors are a novel type of biosensor that employs an aptamer as the biological recognition element and possesses a high affinity to the target. Aptamers are oligonucleotides that can specifically bind target molecules based on a combination of hydrogen bonding, electrostatic interaction, van der Waals forces, and their three-dimensional conformation [215]. Many DNA or RNA aptamers with high affinity and specificity have been identified with various targets, including proteins, peptides, amino acids, antibiotics, small chemicals, viruses, whole or parts of cells, and even metal ions. Aptamers are DNA or RNA fragments derived from selection experiments that have a high affinity for a given target [62,216]. The selection of the appropriate aptamer for a given molecule is accomplished in vitro via a systematic evolution of ligands by exponential enrichment (SELEX) process from libraries containing random oligonucleotide sequences. Based on strong interaction, such as π–π stacking, hydrogen bonding, and electrostatic force that can be formed between special functional groups on organic linkers of MOFs and negative charge of nucleic acid, sequences of a series of biosensors have been developed for food safety analysis [204,210]. For incidence, Chen et al. developed an electrochemical biocode based on a nanoscale MOF for the simultaneous detection of multiple antibiotics with a low DL [205].

### 3.4. MOF-Based SERS Sensing Method

Because of its strong dominance of the interaction and distance targets and nanoparticles, surface-enhanced Raman scattering (SERS) has been widely used in food safety analysis. Because SERS can provide a wealth of structural information, it has been widely used for molecular identification and structural characterization of various compounds, also known as molecular fingerprinting [217]. The widely accepted mechanism for SERS signal enhancement is dominated by electromagnetic field enhancement, which attributes to the localization of surface plasm resonance (LSPR) or hot sport of noble metals and the physical or chemical adsorption of analytes to the surface for metal-analyte charges-transfer production. The adsorptive interaction between suspended colloids and the target in solution is dependent on the slow diffusion of analyte from the bulk solution to the surface of metal nanoparticles (NPs) for the facilitation of molecule-metal interactions [218].

However, various molecules exhibit slow affinity or no affinity for the LSPR areas, limiting the use of SERS techniques. Therefore, much effort has focused on the functionalization of NPs (Au and Ag) with viologen dictations, cyclodextrin, alkanethiolate tri (ethylene glycol), and cysteine, etc., aiming to improve the affinity of the target to the metal surface [219]. The metallic colloids, on the other hand, are easily aggregated, resulting in precipitation into solution and loss of SERS signals [219,220]. Therefore, significant efforts have been made to protect NPs by coating them with organic or inorganic shells such as polymers, transition-metal materials, carbon, and mesoporous silica for mechanical stability and improved signal reproductivity [221]. The majority of these shells are made up of disordered and amorphous structures, and the diffusion of molecules to the metal core is limited. It would be advantageous to develop a SERS detection element with excellent stability and enhanced analyte-metal interactions [222]. Yuling Hu (2014) created a sensitive SERS substrate by embedding AuNPs within MIL-101 using the unique properties of MOFs (high surface areas, tailorable chemistry, and uniform and tunable nanostructured cavities). The SERS substrate that was created was used to detect Rhodamine 6 G and benzidine with detection limits (DL) of 41.75 and 0.54 f mol, respectively. Furthermore, the use of novel SERS in the quantitative analysis of organic pollutant P-phenylenediamine in water and tumor marker alpha-fetoprotein in human serum demonstrated good linearity of 1.0–100.0 ng/mL and 1.0–130.0 ng/mL, respectively [218].

## 4. Use of MOF-Based Sensors for Food Safety Analysis

Because food safety is a major global concern, there have been concerted efforts to develop highly efficient technologies for monitoring food quality and ensuring food safety. In recent years, there has been a surge in the use of novel analytical techniques for determining food quality and safety. MOFs have received a lot of attention in this field because of their high porosity, structural diversity, and tailorability. In the section that follows, we discuss notable works in the detection and sensing of food safety analysis using MOFs and MOF-based materials. The MOF-based method’s application is explicitly dependent on their structural attributes. Understanding the precursor components and synthesis methods reveals that MOFs are mesoporous, have a high specific surface area, open metal sites (OMS), a low framework density, tailorable luminescent properties, and are easily conjugated with the guest species. These properties, in turn, account for a wide range of functional properties such as luminescence, conductivity, chromogenicity, and optical properties. The architectural design of MOFs can lead to the construction of new, highly efficient MOFs with specific applications by taking into account the functional property of the structure and capability relationship. The most commonly reported strategies for food safety analysis, as discussed in previous sections, are luminescent and electrochemical approaches.

### 4.1. Detection of Pathogenic Bacteria

Food-borne disease is one of the most major public health problems, and failure to detect foodborne pathogens may lead to terrible consequences. Biological hazards cause various infectious diseases [223]. Detection and identification of pathogens is the best way of clinically diagnosing them. Microorganisms are widely distributed in nature and in different ecosystems such as water, soil, air, oceans, food, skin, and the intestinal tracts of humans and animals. While many microorganisms are indispensable in ecosystems, some of them are responsible for diseases [1]. Bacteria that are commonly responsible for outbreaks in different countries include *Escherichia coli*, *Salmonella*, *Vibrio chorea*, *Shigella, Listeria monocytogenes*, *Staphylococcus aureus*, *Bacillus aureus*, *Clostridium perfringens*, *Campylobacter jejuni*, and *Legionella*. All of these pathogens can cause gastrointestinal disease, fever, diarrhea abdominal cramps, vomiting, and nausea and lead to the deleterious consequences on the global economy and human health. Significant improvements in the disinfection in food safety have been achieved such as rigorous, good manufacturing practices and good agricultural practices, but the results of food-borne pathogenic microorganism control are still not optimistic. Therefore, routine monitoring of the quality and safety of food is important for public health [4,58,224].

Based on the diverse structural configuration and exciting optical proprieties, MOFs have attracted huge attention for biosensing applications [225]. LMOFs have a number of distinct advantages over other materials, including crystallinity, nano-to-micro sized structures, stable fluorescence over time and temperature, and readily available functional groups for the conjugation of biorecognition species [225]. For the first time, Neha et al. (2019) reported a non-toxic, biocompatible, and water-stable luminescent biosensor MOF with NH2-MIL-53(Fe) as a fluorescent marker. According to the pre-existing literature, NH2-MIL-53(Fe) was solvothermally prepared [226]. The mixture of FeCl_3_.6H_2_O and NH_2_-BDC in deionized water (same concentration of 5 mmol) were prepared and transferred into sealed containers then treated with autoclave heating at 150 °C over a period of 3 days. The synthesized MOF (NH_2_-MIL-53) was filtrated, washed twice with water and ethanol, then dried at 70 °C [226]. The conjugate of antibody- NH_2_-MIL-53 (2 mg mL^−1^) was prepared in flowing way: NH_2_-MIL-53 MOF containing amine functional group was mixed with antibody solution (0.1 mg mL^−1^ into the mixture of 0.1 M PBS, 10 nM EDC, and 5 mM NHS), then incubated at 4 °C overnight for amide linkage formation. The Ab-NH_2_-MIL-53 conjugate was washed with PBS buffer (three times) to remove any unbound Ab or MOF particles. Complex anti-*S. aureus* antibody-MOF (Ab-NH_2_-MIL-53) has been applied to detect different samples, including real samples. The specific binding of complex to bacteria has led to the reduction in fluorescence intensity at the corresponding number of bacteria in solution. Thus, it has given Ab-NH2-MIL-53 biosensors the ability to detect 85 CFU mL^−1^ as DL with over a wide concentration range 4 × 10^2^–4 × 10^8^ CFU mL^−1^ of *S. aureus* [226].

Bacteriophages are a type of bio-recognition element. Bacteriophages are obligate host living parasites that use their tail proteins to recognize the host bacterium with high strain specificity [227]. Therefore, bacteriophages can be used in the development of biosensors with the added benefits of sensor stability in various environmental conditions of pH and/or temperature change, the ability to differentiate viable and dead cells, no sample pre-processing being required, self-signal amplification, and low production cost [227]. Interestingly, bacteriophages can be stable in dried conditions, giving them a distinct advantage over other biomolecules used in biosensor development [228,229]. Neha et al. (2016) designed a bacteriophage-MOF opto-sensor for rapid detection of *Staphylococcus arlettae* [196] by taking into account the micro-size of the bacteriophages (100–200 nm) [196].

A host-specific bacteriophage to *S. arlettae* has been conjugated to the surface of metal-organic framework (IRMOF-3) using the covalent attachment. IRMOF-3 was prepared at room temperature condition as reported in the literature by magnetically stirring mixing Zn (NO_3_)2.6H_2_O (16 mmol) and 2-amino terephthalic acid (8 mmol) in DMF solution with a total volume of 160 mL. The triethylamine (64 mmol) was slowly added, which led to instant white precipitates formation. Produced IRMOF-3 was collected by filtration and washed three times with DMF solvent then immersed into CH_2_Cl_2_ over 72 h, and the product was finally dried under vacuum condition at 70 °C [196]. The highly specific bacteriophage was isolated and purified according to the literature, and the maintained stock solution concentration was 10^8^ PFU mL^−1^ [196]. Bioconjugation of IRMOF-3 with the *S. arlettae*-specific bacteriophage process was achieved by adding 2 mg mL^−1^ of IRMOF-3 into 10 mL Saline Magnesium buffer (pH 7.5) mixed with 2 mL of 25% glutaraldehyde, followed by incubation for 30 min at room temperature; thereafter, 3 mL of bacteriophage solution was added. The function of glutaraldehyde was to catalyze the conjugation reaction of IRMOF-3 with the *S. arlettae*-specific bacteriophage. Unbounded or loosely bound moieties were separated by washing bacteriophage-IRMOF-3 complex twice with Tris-buffer. The purified probe was stored at 4 °C for further usage after drying in vacuum condition [196]. The detection of *S. arlettae* was accomplished by observing changes in the photoluminescence intensity of the probe as it interacted with various concentrations of bacterium solution. The proposed bacteriophage-based biosensor had a detection range of 102–1010 CFU mL^−1^ and a DL of 100 CFU mL^−1^ [196].

**Figure 4:**
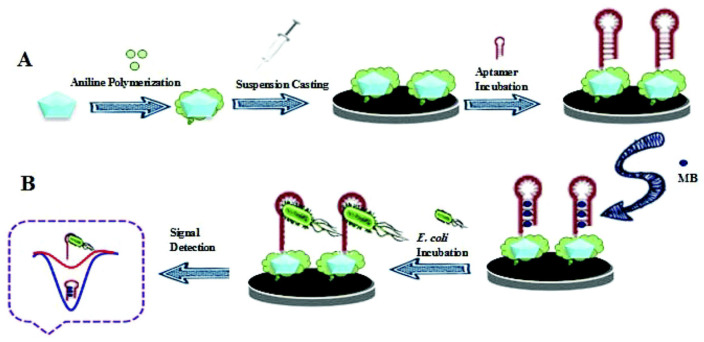
Schematic diagram illustrating (**A**) aptasensor fabrication and (**B**) *E. coli* O157:H7 detection [232]. Copyright permission has been obtained.

Based on the advantages of electronic (sensitivity, portability, and ease of preparation as key devices), MOFs (high porosity, effective surface area, thermal and chemical stability, and tunable pores sizes), and aptamer (high selectivity, specificity, cheap, and easy to select by SELEX process), Saeed and Saba (2018) reported an electrochemical MOF-based biosensor for detection of *E. coli* 0157:H7. The synthesis of CU3(BTC)2(HKUST-1) and Cu-MOF/PANI nanocomposites was carried out in accordance with previously published studies, with some modifications [230,231]. The glassy carbon electrode (GCE) was polished with alumina slurry (0.1 M) with a polishing cloth, rinsed with water, and then sonicated in ethanol for 5 min to create the MOF-aptamer biosensor. Figure 4 depicts the synthesis of the complex PANI/MOF/GCE [232]. Aptamer -NH_2_ groups were covalently linked to PANI/MOF -NH_2_ groups with GA. In fact, the PANI/MOF surface provided a large number of free amine groups for aptamer immobilization. The developed biosensor was monitored using the cyclic voltammetry (CV) and electro-chemical impedance (EIS) techniques. As a result, using methylene blue (MB) as an electronical indicator, differential pulse voltammetry (DPV) was used to monitor and quantify the interaction between the aptamer and *E. coli* 0157:H7. The recorded current change (in reduction) of MB was an analytical signal indicator of the relationship with the logarithm of *E. coli* 0157:H7 concentration in the detection range of 2.1101–2.1107 CFU mL^−1^ with DL of 2 CFU mL^−1^ [232].

### 4.2. Detection of Heavy Metals

Environmental contamination by heavy metals has been an important issue worldwide. Some of these heavy metals are even not biologically essential, including Pb, Hg, and Cd. Among these heavy metals, Hg is an effective neurotoxin owing to its accumulation in the vital organs and tissues; additionally, its binding to the sulfur-containing proteins and enzymes destroys important cell functions which can lead to disease [233]. Heavy metals can cause toxicity and are a source of severe damage to ecosystems, cause economic losses, and negatively impact the food chain and health due to their lack of biodegradability. There are many ongoing studies on the development of different techniques for the detection of heavy metals at trace levels in the environment, food products, and water, as well as in living organisms [234]. Different studies have been conducted to develop several new methods for heavy metals detection at trace levels. A stripping voltammetric method was developed, and other methods such as mass-spectrograph, plasma-induced spectrum, atomic fluorescence spectrometry, and ultraviolet-visible spectrometry were subsequently developed [235].

While these methods each have advantages, there are also disadvantages, such as complicated procedures for sample pre-treatment, expensive instruments that are operated by professionals, and being time-consuming. In order to overcome these deficiencies, different attempts have been made to establish better sensors for rapid and easy detection of metals including the MOF-based detection method [236,237,238]. Therefore, this study discusses the recently developed MOF-based detection method for sensing heavy metal in water and food. Scientists recently reported that organic linkers on MOFs contain special functional groups that could serve as a source of stacking, hydrogen bonding, and electrostatic interactions with negatively charged molecules. As a result, MOFs can be used as a recognition element in biosensors for small ions or nucleic acid molecules [239]. Furthermore, considerable effort has been expended in obtaining supporting materials with broad properties such as high-water stability, biocompatibility, adsorbent capability, and electrochemical activity for the application of MOF in food safety analysis [238].

**Figure 5:**
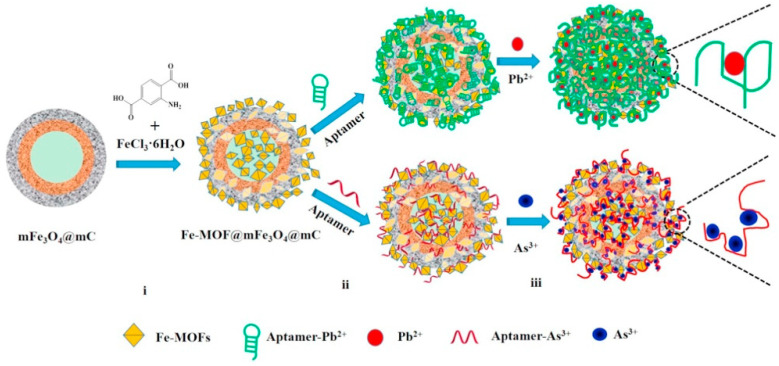
Preparation process for Fe-MOF@mFe_3_O_4_@mC nanocomposite and its related aptasensor for detection Pb^2+^ and As^3+^ via electrochemical techniques, including (i) the preparation of Fe-MOF@mFe_3_O_4_@mC nanocomposite, (ii) the immobilization of the aptamer strands, and (iii) the determination of the heavy metal ions [238]. Copyright permission have been obtained.

Zhang et al. (2017) created a new core-shell nanostructured of Fe-MOF@mFe_3_O_4_@mC with an inner cavity and an orderly mesoporous opening structure for incidence. The developed core-shell was attached to porous structure aptamer sequences for heavy metal detection (Pb^2+^ and As^3+^). The steps of biosensor fabrication were involved, including the preparation of Fe-MOF@mFe_3_O_4_@mC, the immobilization of aptamers, and the detection of Pb^2+^ and As^3+^. In the presence of the hallow Fe_3_O_4_@mC nanocapsules, the core-shell nanostructured of Fe-MOF@mFe_3_O_4_@mC were hydrothermally prepared, with FeCl_3_ acting as the precursor and 2-amino-terephthalic acid acting as a linker, obtained after calcination of hallow Fe_3_O_4_@C nanocapusules, which were synthesized from core-shell SiO_2_@Fe_3_O_4_@C spheres with SiO_2_ removed. The intensive binding between Fe-MOF and the aptamer sequence could generate a high immobilization force for the aptamer sequences due to supramolecular stacking and hydrogen-bonding interactions. When Fe-MOF is added to a solution containing aptamers, the aptamers tend to approach the surface of the Fe-MOF (Figure 5). As a result, the designed strategy has proven to be a suitable analyzer for traces analyte by detection of heavy metal (Pb^2+^ and As^3+^) in river water and blood serum, with a detection range of 0.01 to 10.0 nM and estimated DL of 2.27 and 6.63 PM toward detecting Pb^2+^ and As^3+^, respectively [238].

Based on various advantages of facile, ecological MOF preparation such as simple instruments, the occurrence of reaction at atmospheric pressure, and convenient reaction process, for the first time, Wang et al. (2015) fabricated a cauliflower-like MIL-100(Cr). After preparation, MIL-100(Cr) was confirmed by FT-IR, XRD, SEM, and XPS to apply in detection of heavy metal ions (Cd^2+^, Pb^2+^, Cu^2+^, and Hg^2+^) in aqueous solutions at trace amounts [240]. In the concentration range of 0–10 M, a correlation coefficient of Cd^2+^, Pb^2+^, Cu^2+^ and Hg^2+^ were 0.991, 0.9868, 0.989, 0.997, respectively with DL of 4.4 × 10^−8^ mol L^−1^ for Cd^2+^, 4.8 × 10^−8^ mol L^−1^ for Pb^2+^, 1.1 × 10^−8^ mol L^−1^ for Cu^2+^, and 8.8 10^−9^ mol L^−1^ for Hg^2+^ [240]. Ionic luminescent metal-organic framework (ILMOF) is a new LMOF composed by a charged hybrid material of atoms and organic ligand which contains advantages electrification and intrinsic properties of MOF [241,242,243]. Based on the higher affinity of Hg^2+^ to the nitrogen atoms, Wan et al. (2018) selected [2, 2′:6′, 2″-Terpyridine]-4, 4′, 4″-tricarboxylic acid (TPTC) to design a MOF with organic ligand which contained multiple nitrogen atoms (N) for Hg^2+^ detection. The designed Zn-TPTC MOF was performed in the detection of Hg^2+^ in water with a wide detection range of 10^−6^–10^−4^ M, calculated DL was as low as 3.67 nM [243]. Thus, we have a generalized idea of MOF selection and designing using pore size, anionic frameworks, and multiple N sites in the organic ligand.

### 4.3. Detection of Illegal Food Additives

In recent years, food adulteration has become a public health issue as well as a food safety problem. Sudan dyes have been detected in spice powders, chili sauces, spicy soups, colorful desserts, and even soft drinks [244]. Such illegal synthetic dyes are cheap and easily used as coloring agents to enhance the natural color of products. Adulteration of natural milk with synthetic chemicals is a serious problem for human health [47]. For incidence, melamine (1,3,5-triazine-2,4,6-triamine, C_3_H_6_N_6_) is an industrial chemical compound with high nitrogen content (66% by mass) which used in melamine resins synthesis. Recently, it has been fraudulently added in milk to false a higher level of protein concentration which is evaluated by determination of nitrogen concentration with the Kjeldahl method. The addition of melamine into food products has been a cause of serious diseases and many babies and children were intoxicated [245,246]. Therefore, the detection of illegal additive compounds at trace levels would be advantageous. HPLC coupled with ultraviolet (UV), thin-layer chromatography (TLC), diode array (DAD), and ELISA are still used for detecting toxins and food illegal additives. However, all these methods require complicated and expensive sample pre-treatment, skills of a trained operator, and expensive equipment with low analyte concentration [57,246]. Therefore, the development of a reliable and sensitive detection method which can realize real-time and convenient detection of food adulteration of great importance.

**Figure 6:**
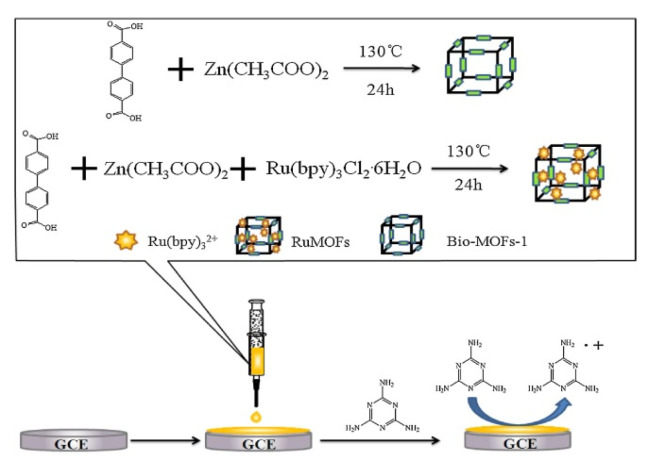
Design process of the ECL sensor for melamine detection in dairy products [247]. Copyright permission has been obtained.

Based on high sensitivity, rapid response, wide linear range, good controllability, low background, and low DL, various scientists have reported on the application of the ECL method as an analytical tool for food safety detection. However, there have been few reports of the application of MOF into ECL systems, because of a lack of redox and luminescence properties in organic ligands of reported MOFs. To overcome this problem, Feng, et al. (2018) designed and synthesized a doped MOF with Tris(2,2′-bipyridyl) dichlororuthenium (II) (Ru (3^2+^) for melamine detection in daily products. The main used building block units were the anionic bio-MOFs-1 [Zn_8_(ad)_4_(BPDC)_6_O.2Me_2_NH_2_,8DMF,11H_2_O] (ad = adeninate; BPDC = biphenyl carboxylate; DMF = dimethylformamide) with columnated zinc-adeninate as a secondary building unity composed of apex-sharing zinc-adeninate octahedral cages, while the Ru(bpy)3^2+^(luminescent cationic) were doped into the MOF and their original electro-chemical and luminescent properties were preserved. The ability of Ru(bpy)3^2+^ to react with amides on melamine (1,3,5-triazine-2,4,6-triamine) has attracted more attention as a potential application in the synthesis of MOF-ECL based method for melamine detection (Figure 6). Under optimum conditions, the ECL intensity was proportional to log (melamine concentration) in the wide detection range of 10^−10^–10^−4^ with DL of 3.8 × 10^−11^ M [247]. The designed method was successfully applied in milk and infant formula powder melamine detection recoveries in the range 98–104% and 95–103%, respectively, obtained from spiked samples [247].

### 4.4. Detection of Natural Toxins in Food

Food is only one source of nutrients but may also contain potentially harmful natural toxic substances to humans including mycotoxin, a bacterial toxin, animal biotoxin, neurotoxin, and phytotoxin. The toxicological effect of some of these substances can be acute even at a very low dose. Therefore, many classical methods have been developed for toxin detection in food [15,16].

Recently, researchers have been drawn to the combination of MOFs with other superior functional materials such as quantum dots (QDs), polyoxometalates (POMs), polymers, graphene, and carbon nanotubes (CNTs) because this technology may present advantages of their merits while mitigating their shortcomings [248,249,250]. On the other hand, two-dimensional (2D) layered materials like graphitic-phase carbon nitride (g-C_3_N_3_) have been widely applied in sensing, drug delivery, and imaging, and they can be regarded as N-substituted graphite in a regular fashion [251,252].

**Figure 7:**
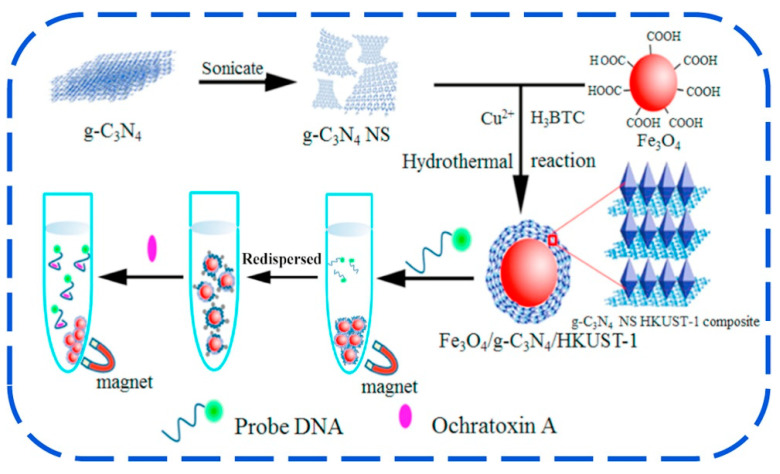
Schematic diagram representing principle of the biosensor based on Fe_3_O_4_/g-C_3_N_4_/HKUST-1 to detect OTA [199]. Copyright permission has been obtained.

However, the affinity of g-C_3_N_4_ for aptamer is low, which may result in aptamer desorption from the material’s surface without the addition of target, lowering the sensor’s stability [253]. To surmount this situation, Hu and his colleagues (2017) referred to Zhang et al.’s (2014) work (the combination of MOF with Carbone nanotube) to combine HKUST-1 with g-C_3_N_4_ to form the g-C_3_N_4_/HKUST-1 complex, where g-C_3_N_4_ were acting as hydrophobic protection of HKUST-1 from water molecules [199,254]. The Fe_3_O_4_ was introduced for lowering the background, then the formed Fe_3_O_4_-g-C_3_N_4_/HKUST-1 composites were to be used in the development of aptasensor for OTA detection in a corn sample as described in Figure 7. The developed composites have a high adsorption capacity for dye-labeled anti-OTA aptamers and can completely quench the dye’s fluorescence via a photoinduced electro transfer (PET) mechanism. In the presence of OTA in solution, it can bind with high affinity to the aptamer, resulting in the leasing of dye-labelled aptamer from quencher (Fe_3_O_4_-g-C_3_N_4_/HKUST-1) and an increase in fluorescence. The aptasensor’s fluorescence intensity had a linear relationship with the OTA concentration in the range of 5.0–160.0 ng mL^−1^, with a DL of 2.57 ng mL^−1^ [199].

Based on LMOF’s advantages of having an easy-to-functionalize surface and tunable porosity which can promote feasible guest-host interactions, for the first time, LMOF for very fast and sensitive fluorescence-based mycotoxin were developed for OTA detection [255]. Synthesis of Zn(bpdc)_2_(tppe) (LMOF-21) started from ligand 1,1,2,2-tetrakis(4-(pyridine-4-yl) phenyl) ethane(tppe) synthesis based on a reported process [256] where solid 1,1,2,2-tetraphenylethene (tpe) reacted with liquid bromine to produce 1,1,2,2-tetrakis(4-bromophenyl) ethene (Br_4_-tpe) with recrystallization purification in dichloromethane/methanol. Br_4_-tpe and pyridine-4-4bronic acid were reacted in catalysis of palladium (acetate) for the attachment of the pyridine moiety to the tpe moiety. Chloroform and column chromatography were used in the extraction and purification of the product, respectively. Thereafter, a mixture of Zn(NO_3_)_2_·6H_2_O (0.015 g, 0.05 mmol), biphenyl,-4,4′-dicarboxylic acid (H_2_bpdc, 0.012 g, 0.05 mmol), tppe (0.013 g, 0.02 mmol), N,N-dimethylacetamide (DMA, 8 mL), dimethyl sulfoxide (2 mL), and isopropyl alcohol (2 mL) was added in a 20-mL glass vial. After ultrasonication mixing, the glass vial was sealed and kept at 150 °C for 24 h and then cooled down to room temperature for the filtration process. Optic proprieties evaluation of LMOF-241 proved its ability of blue-green emitting LMOF with an exceptionally high internal quantum yield (92.7%). The developed LMOF was successfully applied in mycotoxin detection via quenching mechanism with high optical selectivity and the calculated DL was 46 ppb [255].

### 4.5. Detection of Drug and Pesticide Residues

Pesticides and veterinary drugs are an important tool in agro-business to control insects, weeds and diseases and improve crop and livestock yield by minimizing losses. However, many scientists proved the harmful impact of veterinary drugs and pesticides to the environment as well as to humans via food consumption [257,258]. Utilization of veterinary medicines, especially antibiotics, plays an important role in animal feed production through treatment and disease prevention and growth promotion as well [259]. However, various scientific reports proved that the use of antibiotics in animals can result in antibiotic residues in foodstuffs such as milk, eggs, and meat. These residues may cause side effects such as the transmission of antibiotic-resistant bacteria to humans, immunopathological effects, allergy, mutagenicity, nephropathy, hepatotoxicity, reproductive disorders, bone marrow toxicity, and carcinogenicity through human conception [259,260,261]. On other the side, the routine utilization of pesticides in modern agriculture has increased agricultural crop yield. However, it has proved that pesticides can be serious sources of food safety hazards [262,263]. Therefore, the detection of pesticides and drug residue at trace amounts in food is necessary.

During the last decade, different studies have been carried out to develop different analytical techniques for pesticides and drugs residue detection, including capillary electrophoresis, surface plasmon resonance, HPLC, microbiological methods, immunoassays, and electrochemical immunosensors [60]. Usually, these methods are very expensive, time-consuming, and require expensive equipment and highly-trained technicians. Recently, with featuring tunable intriguing structures, permanent porosity, and structural flexibility, MOFs has been used for pesticide and drugs residue detection in food and the environment [264,265,266]. Therefore, different authors have reported and reviewed the application of MOFs in the detection of pesticide and drug residue detection in food and the environment. For incidence, Vikrant et al. (2018) highlighted recent advancements in MOF-based sensing techniques for pesticides with emphasis on the description of sensing principals of MOFs along with areas of practical applications in pesticide detection [266]. Therefore, this subtitle of the application of MOFs in the detection of pesticide and veterinary drug residues focused on the recent developed MOFs-based analytical techniques for drugs residue detection in food.

LMOFs have tunable intriguing structures, permanent porosity, and intense fluorescence, which has sparked a lot of interest recently for their potential use in fluorometric chemosensors. As a result, Zhou and her coworkers (2018) reasoned that tetracycline (TC) detection and absorption could be accomplished through electron/energy transfer and specific host-guest interactions between TC and MOF by carefully selecting the component metal ions and organic ligands. As a result, a highly stable luminescent zirconium-based MOF (PCN-128Y) for the detection and removal of TC in water was created. PCN-128Y was constructed by tetraphenylethylene (TPE)-based ligand H4ETTC (which can serve as fluorophore and its mission can be quenched by TC) and Zr_6_ clusters (with coordination sites terminal OH/H_2_O which can facilitation of TC absorption). The synthesis of PCN-128YZrCl_4_ started from mixing ultrasonically of H_4_ETTC (60 mg, 0.072 mmol) and trifluoroacetic acid (0.08 mL) in Pyrex tube contained 8 mL DMF, then was heated at 120 °C for 48 h. The harvested white, solid product was transferred into a mixture of DMF and HCl, then stirred at 100 °C in an oil bath for 12 h. The centrifugation separation was performed and the product washed by with DMF and acetone five times. The yellow product of PCN-128 was obtained after centrifugation and drying at 70 °C for 6 h under vacuum condition. The application of PCN-128 in TC sensing was successful with significant luminescence quenching (0.1 mM quenched 90% of PCN-128 luminescence) in 1 min [267].

However, the high selectivity was not well achieved where tested antibiotics presented 5–40% fluorescent quenching capacity except TC. Therefore, a nanoscale luminescent MOF (ln-sbdc) was synthesized from In^3+^ (metal ion) and ligand of *trans*-4,4-stilbenedicarboxylate (sbdc2^−^) for recognition of TCs over a series of other kinds of antibiotics in food and the environment [264]. The synthesis of ln-sbdc MOF was performed at room temperature by mixing InCl_3_ with H_2_sddc in the DMF-H_2_O solvent. Synthesized MOFs which were successfully applied in the detection of tetracycline series antibiotics included tetracycline, chlortetracycline, and oxytetracycline with DLs of 0.28–0.30 μM. The selectivity test showed that the other eight tested kinds of antibiotics did not cause an equable change in its emission [264].

The application of MOFs in SERS technology has provided a new route for pesticide detection by embedding NPs with MOFs. Cao (2017) successfully embedded AUNPs into MOFs (MOF-199, Uio-66, and Uio067) for SERS enhancement. The synthesized AuNPs-MOF-199, AuNPs-Uio-66, and AuNPs-Uio-67 composites exhibited excellent SERS activity. The application of developed approaches to the detection of acetamiprid was successfully achieved with DL of 0.02 μM, 0.009 μM, and 0.02 μM [268].

### 4.6. Persistent Organic Pollutants (POPs)

Persistent organic pollutants (POPs) are various classes of toxic organic compounds that can persist in the environment and have the potential to bio-accumulate in biological organisms, resulting in a variety of health effects in both animals and humans. As a result, POPs have been classified as important environmental and food contaminants due to their resistance to degradation, ability to travel long distances by air, water, and sedimentation to new environmental media located far away from the original released source [269]. These POPs have a long half-life spread in the environment for a long period of time, which may accumulate and increase significantly in the food chain as well as in the living organism and have adverse effect on human beings and the environment in general [116,270]. Therefore, it is greatly important to establish simple, rapid, low-cost and sensitive analytical methods for trace detection of POPs in food and the environment. Recently, the conventional method has been developed and applied to POPs detection [271]. However, these methods can provide reliable analytical results but generally require complicated sample preparation processes and skilled personnel. Therefore, is urgent to develop new methods that are highly efficient and easy to perform for detection POPs.

Based on remarkable luminescence properties of lanthanide MOFs (Ln-MOFs) and their applications as luminescent sensors, a new Ln-MOF 1 was synthesized for detection of polychlorinated benzenes including 1,2,4-trichlorobenzene (1,2,4-TCB), 1,2,3,4-tetracholobenzene (1,2,3,4-TCB), 1,2,3,5-tetracholorobenzene (1,2,4,5-tcb), pentacholorobenzene (PeCB), and hexachlorobenzene (HCB). The synthesis of [(Eu_2_(L)_3_(DMF)_2_].DMF.MeOH}_n_ (Ln-MOF 1, H2L = 5-(4H-1,2,4-triazol-4-yl)benzene-1,3-dicarboxylic acid, MeOH = methanol, DMF = N, N-dimethylformamide) was performed through a coordination symmetry approach [272]. The systematical luminescence studies showed that Ln-MOF 1 have a quenching ability on detecting polychlorinated benzenes series, and the increasing of the chlorine atoms number on benzene corresponded to decreasing luminescent intensity [272].

## 5. Conclusions and Future Research

MOFs are functional materials, which present unique physical and chemical properties that are not available with other conventional porous materials, namely zeolites and activated carbons. The structural modularity with post-synthetic functionality and exceptionally controlled porosity make MOFs ideal candidate materials to be used in food safety analysis. They also offer enormous opportunities for sensing and absorption in various areas such as medicine, agriculture, environmental sciences, bio-analytical fields, and food safety. Various MOFs have been designed and synthesized and applied in food safety analysis. To enhance the stability and sensitivity of MOFs in complex samples, post-synthetic modifications have been focused on the functionalization of signals emitted by nanomaterials such as gold nanorods or gold nanoparticles, quantum dots, silver nanoclusters, and magnetic beads and incorporation of biomolecules.

These post-synthetic functionalizations have significantly contributed to MOF-based sensor design for food safety analysis. Therefore, the future of MOF-based sensor designs for food safety analysis would strongly depend on the reported investigations, which are still in need of improvement. The majority of studies revealed that MOFs are efficient food safety sensors, but there are some specific cases presenting the opposite conclusion, which may cause by differences in experimental conditions and specific interactions with surface functional groups. In this aspect, stringent MOF stability specificity and selectivity are the main features required to obtain an analytical sensor capable of meeting food safety prerequisites. Furthermore, the food industry requires the availability of cheap and easy-to-manipulate analytical sensing tools at every single step of the food chain, even in remote areas with good sensing capacity. In this regard, an active collaboration between different scientific disciplines may overcome the technical hurdles and improve the exciting MOF-based sensors for food safety analysis. The multipipeline-scientific integration approach will provide rational and practical designs of MOF-based sensing methods, which are easy to manipulate at point of care, raw-cost, portable, robust, and sensitive with multianalyte sensing capacity and the ability to remove and absorb contaminant in food without any contamination.

## Figures and Tables

**Table 2:** Sensing application of MOF-based Electrochemical Sensing for food safety analysis.

	MOF Synthesis Process							Application in Detection			
Target Food Hazards	MOF	Modification	Metal/Metal Cluster Source	Ligand Source	Solvent	Time (h)	Temperature (°C)	Real Samples	Linear Range	DL	Reference
Citric acid	MIL-101(Fe)	CPE	FeCl_3_·6H_2_O	Terephthalic acid	DMF	20	110	beverage	5 × 10^−6^– 100 × 10^−6^ M	44 × 10^−6^ M	[133]
Paracetamol(PC) and caffeine(C)	MOF-199 (HKUST-1)	GCE	Cu(NO_3_)_2_·3H_2_O	H_3_BTC	Ethanol, water and DMF	8	180	pharmaceutical tablets		C: 1.2 μM	[134]
										PC:1.3 μM	
Clethodim	MIL-125(Ti)/TiO_2_	GCE	TBT	H_2_BDC-NH_2_	DMF and methanol	20	150	Soil	0.2–25 (μmol L^−1^)	0.03 (μmol L^−1^)	[135]
2,4,6-trinitrophenol	C-BTC MOF	GCE	Cu(NO_3_)_2_	H_3_BTC	DMF and ethanol	12	120	water	0.2–10 μM	0.1 μM	[136]
Metronidazole	ZIF-67 MOF	GCE	Co(NO_3_)_2_·6H_2_O	2-methyldinazole	water	5	90	water	0.5–1000 μM	0.05 μM	[137]
Hydrazine	ZIF-67	CPE and AgN	Cobalt nitrate hexahydrate	2-methylimidazole	methanol	24	room temperature (RT)		4–326 μM	1.45μM	[138]
Parathion	ZIF-8MOF	CPE	Zn(NO_3_)_2_·6H_2_O	2-methylimidazole	methanol	24	RT	vegetables	5.0–700 μg/L	2.0 μg/L	[139]
Malathion	Cu-BTC MOF	CUO	Zn(NO_3_)_2_·6H_2_O	H3BTC	Acetic acid and TEA	24	85	Chinese cabbage	10^−10^–1.0 × 10^−5^ mol L^−1^	8.6 × 10^−11^ mol L^−1^	[140]
estradiol	Cu-BDC MOF	CPE	Cu(OAc)_2_·H_2_0	H_2_BDC	DMF and water	2	RT	water	5 to 650 nM	3.8 nM	[141]
Malachite green	Ag/Cu MOF	GCE	Cu(NO_3_)_2_·3H_2_O and	BTC	Water and ethanol	14	120	fish	10–140 nM	2.2 nM	[142]
Hydroquinone (HQ) and catechol (CT)	FJU-40-H/NH2 MOFs	NPC	Zn(NO_3_)_2_·6H_2_O	BDC and Trz	Water, ethanol and DMF	24	85	water	HQ = 1–70 µmol L^−1^	HQ = 0.18 µmol L^−1^	[143]
									CT = 1–100 µmol L^−1^	CT = 0.31 µmol L^−1^	
Cd (II) and Pb (II)	Bi/MIL-101 (Cr) MOF		CrCl_3_·6H_2_O	TPA	water	20	200	water	Cd (II) and Pb (II) = 0.1 ~90 μg L^−1^	Cd^2+^:0.06 μg L^−1^	[144]
										Pb^2+^: 0.07 μg L^−1^	
Nitrite	NH_2_-MIL-101(Cr) MOF	SPCE	Cr (NO_3_)_3_·9H_2_O	2-aminoterephthalicacid	NaOH	16	160	sausage and pickle	5.00 × 10^−6^–1.5 × 10^−4^ nM	1.3 nM	[145]
tetrabromobisphenol	PCN-222(Fe) MOF	acetylene black	ZrCl_4_ and Fe-TCPP	benzoic acid	DMF	48	120	water	0.001–1.0 μmol L^−1^	0.57 nmol L^−1^	[146]
Bisphenols (BPs: BPE, BPF, BPA, BPB, and BPZ)	Cu-MOF	GCE	copper nitrate trihydrate	Triethylenediamine and benzene dicarboxylic acid	DMF	36	120	wastewater	BPE: 5.0 × 10^−8^ to 3.0 × 10^−6^ nM	BPE:15 nM	[147]
									BPF: 5.0 × 10^−8^ to 3.0 × 10^−6^ nM	BPF:16 nM	
									BPA: 5.0 × 10^−8^ to 3.0 × 10^−6^ nM	BPA:13 nM	
									BPB: 1.25 × 10^−7^ to 8.0 × 10^−6^ nM	BPB:56 nM	
									BPZ: 2.5 × 10^−7^ to 5.0 × 10^−6^ nM	BPZ:33 nM	
Nitrite	Cu-MOF (MOF-14)	CPE	Cu(OH)_2_	H_3_BTB	DMF, DMSO, DW and HNO_3_	48	100	lake water	50 nM–717.2 μM	30 nM	[148]
chloramphenicol	IRMOF-8	GCE	zinc nitrate hexahydrate	2,6-naphthalenedicarboxylic acid	DMF	20	120	honey	1 × 10^−8^–1 × 10^−6^ mol L^−1^ and 1 × 10^−6^–4 × 10^−6^ mol L^−1^	2.9 × 10^−9^ mol L^−1^	[149]
nitrite	Cu-MOF	GCE	CuCl_2_	PVP	Water, NaOH and ascorbic acid	5 min	RT	water	0.1–4000 and 4000–10,000 μM	82 nM	[150]
hydroquinone (HQ) and catechol (CT).	Cu-MOF-199	GCE and SWCNTs	Cu(NO_3_)_2_·3H_2_O	H_3_BTC	DMF and ethanol	12	120	water	HQ: 0.1 to 1453 μmol L^−1^	HQ: 0.08 μmol L^−1^	[151]
									CT: 0.1–1150 μmol L^−1^	CT: 0.1 μmol L^−1^	
uric acid (UA) catechol (CT) hydroquinone (HQ)	ZIF-8	GCE	(NO_3_)_2_·6H_2_O	2-methylimidazole	Water and 2-methylimidazole	RT	30 min	water and seawater	UA and CT:0.001–0.3 HQ: 0.001–0.2 mM	UA: 1.4 × 10^−8^ M	[152]
										CT: 2.78 × 10^−7^ M	
										HQ:2.15 × 10^−7^ M	
HQ and CT	Cu-MOF	GCE	Cu(NO_3_)_2_·6H_2_O	H_3_BTC	DW and ethanol	150	24	tap water	HQ: 1.0 × 10^−6^–1.0 × 10^−3^ M	HQ: 5.9 × 10^−7^ M	[153]
									CT: 1.0 × 10^−6^–1.0 × 10^−3^ M	CT: 3.3 × 10^−7^ M	
HQ, CT and RS	UiO-66 MOF	GCE	ZrCl_4_	BDG	DMF and acetic acid	120	24	lake water		HQ: 0.056 μM	[154]
										CT: 0.072 μM	
										RS: 3.51 μM	
bisphenol A (BPA)	Ge-MOF	GCE	Ce(NO_3_)_3_·6H_2_O	1,3,5-H_3_BTC	Water and ethanol	RT	10 min	fresh milk	0.005–50 μmo L^−1^	0.092 μmol L^−1^	[155]
metolcarb	MIL-101	MIP and QCM	Cr(NO_3_)·39H_2_O	terephthalic acid (TPA)	HF and DDW	220	8	pear juice	0.1–0.9 mg L^−1^	0.0689 mg L^−1^	[156]
Methamidophos (omethoate)	MIL-101(Cr)	GO	(Cr(NO_3_)_3_·9H_2_O),	(C_6_H_4_–1,4-(CO_2_H)_2_),	hydrofluoric acid and DDW	200	8	cucumber and kidney bean	1.0 × 10^−7^–1.0 × 10^−12^ and 1.0 × 10^−7^–1.0 × 10^−13^ mol/L	2.67 × 10^−13^ mol/L and 2.05 × 10^−14^ mol/L	[157]

**Table 3:** Sensing application of the MOF-based luminescence chemosensing method for food safety analysis.

	MOF Synthesis							Application in Detection		
Target Food Hazards	MOF	Metal/Metal Cluster Source	Ligand Source	Solvent	Time (h)	Temperature (°C)	Sample	Linear Range	DL	Reference
UO_2_^2+^	EU-MOF	Eu(NO_3_)_3_·6H_2_O	H_3_TATAB	DMF and H_2_O	72	120		12.5–87.5 μM	0.9 μM	[168]
Berberine hydrochloride (BRH) and tetracycline (TC)	Eu-MOF 1	Eu(NO_3_)_3_·6H_2_O	Terephthalic acid and Hartz	DMF/H_2_O	27	150	urine	BRH = 0.5–320 Μm	BRH = 78 nM	[169]
								TC = 0.05 to 160	TC = 17 nM	
Fe^2+^	SUMOF-7II	LaCl_3_·7H_2_O	2,4,6-tri-*p*-carboxyphenylpyridine(H_3_L2)	DMF, Cyclohexane and water	16	85		16.6 μM	16.6–167 μM	[170]
Clenbuterol	UiO-66 MOF	ZnCl_4_	1,4-benzenedicarboxylic acid	DMF and HCl	16	220	pig and sheep urine	4–40 ng/mL	0.17 μM	[171]
Acetone and Fe^3+^	([Cd_1.5_(DBPT)(DiPyDz)(H_2_O)]· 3.5H_2_O)*_n_ (1)* MOF	Cd(NO_3_)_2_·4H_2_O	H_3_DBPT and 4-DiPyDz	DMA/water	72	130		0.0025–0.025 mM	Acetone = 0.0013% (*v/v*%) Fe^3+^ = 78 ppb	[172]
Sulphonamide Antibiotics	FSC-1 MOF	Zn(NO_3_)_2_·6H_2_O	H_3_L and NaHCO_3_	water	72	130	wastewater			[173]
Cr(VI)	1, H_4_mtb MOF	Eu(NO_3_)_3_·6H_2_O	*N*,*N*-dimethylacetamide	DMA and DW	48	90	water	1 ppb to 300 ppm	DW = 0.56, LW = 2.88, and SW = 1.75 ppb	[174]
Dipicolinic acid (DPA)	Tb-MOF	Tb(NO_3_)_3_·5H_2_O	H_3_BTC	DW and ethanol	1	room temperature		1 nM to 100 μM	0.04 nM	[175]
DPA	Bio-MOF-1	zinc acetate dihydrate	4,4′-biphenyl dicarboxylic acid and adenine	DMF, water and nitric acid	48	130	human serum		34 nM	[176]
3-nitropropionic acid (3-NPA)	Cd(L)·solvent]*_n_* (1)	Cd(NO_3_)_2_·6H_2_O	H_2_L	DMF	72	85	sugarcane		0.135 M	[177]
oridazole antibiotics	CTGU-7 MOF	Eu(NO_3_)_3_·6H_2_O and Na_3_TATAB	DMF	DMF and water	140	72		1 μM to 50 μM	0.8 μM	[178]
quercetin	ZIF-8	Zn(NO_3_)_2_·6H_2_O	2-Hmin	Methanol	RT	1	Ginkgo biloba extract capsules	0–50.0 μM	2.9 nM	[179]
Cr(VI)	Eu-MOFs)	Eu(NO_3_)_3_·6H_2_O	H_3_BTC	DMF and water	100	24	water	2 μM to 100 μM	0.21 μM	[180]
Cu^2+^	Eu-DPA MOFs	Eu(NO_3_)_3_·6H_2_O	DPA	Ethanol	180	73	water	50^−1^ × 10^4^ nM	26.3 nM	[181]
parathion-methyl	znPO-MOFs	Zn(NO_3_)_2_·6H_2_O	H_4_TCPB	DMF	100	48	water	1.0 μg L^−1^–10 mg L^−1^	0.12 μg L^−1^	[182]

**Table 4:** Sensing application of the MOF-based biosensing method for food safety analysis.

			MOF Synthesis							Application in Detection		
Sensing Method	Target Food Hazards	MOF	Biomolecules	Metal/Metal Cluster Source	Ligand Source	Solvent	Time (h)	Temperature (°C)	Sample	Linear Range	DL	Reference
Electrochemical	triazophos (TRS) and thiacloprid (THD)	UiO-66-NH_2_	antibody	ZnCl_4_	2-aminoterephthalic acid	DMF and acetic acid (AC)	8	120	rice	TRS = 0.2–750 ng. mL^−1^	TRS = 0.07 ng. mL^−1^	[197]
										THD = 0.2–750 ng. mL^−1^	THD = 0.1 ng. mL^−1^	
Electrochemical	Malathion	Cu/Ce-BTC MOF	enzyme	Cu(OAc)_2_·3H_2_O and Ce(NO_3_)_3_·6H_2_O (n(Cu):n(Ce)	H_3_BDC	DMF and water	4	100	water	10 fM–100 nM	3.3 fM	[198]
Fluorescent	Ochratoxin A	HKUST-1	Aptamer	Cu(NO_3_)_2_·3H_2_O	H_3_BTC	Water and ethanol	12	120	corn	5.0–160 ng/mL	2.57 ng/mL	[199]
Colorimetric	kanamycin	Fe-MIL-88NH_2_	Aptamer	FeCl_3_·6H_2_O	H2N-BDC	DMF and AC	4	120	milk	0.0005–30 ng mL^–1^	0.2 pg	[200]
	chloramphenicol	Cu-TCPP	Aptamer	Cu (NO_3_)_2_·3H_2_O and CF_3_COOH	PVP and TCPP	DMF and ethanol	3	80	milk and fish	0.001–10 ng mL^−1^	0.3 pg mL^−1^	[201]
photoluminescence	*S. aureus*	NH_2_-MIL-53(Fe)	Bacteriophages	FeCl_3_·6H_2_O	NH_2_-BDC	DW	72	150	pastry cream	40–4 × 10^8^ CFU/mL	31 CFU/mL	[202]
fluorescent	*S. arlettae*	IRMOF-3′	Bacteriophage	Zn(NO_3_)_2_·6H_2_O	2-amino terephthalic acid	DMF	RT	2	river water	10^2^–10^8^ cfu mL^−1^ *S*	100 cfu /mL	[196]
Electrochemical	oxytetracycline	Ce-MOF@COF	Aptamer	Ce(NO_3_)_3_·6H_2_O	Cyanure acid and melamine H_3_BTC	Water and ethanol	90	2	milk, water, and urine	2 × 10^−4^–1.0 nM	35.0 fM	[203]
Electrochemical	ampicillin (AMP)	Co-MOF	Aptamer	Co(NO_3_)_2_·6H_2_O	2-methylimidazole	water	RT	2	water and milk	0.001–2000 pg mL^−1^	0.217 fg mL^−1^	[204]
fluorescence	Bisphenol A	Fe-MIL-88B–NH_2_	Aptamer	FeCl_3_·6H_2_O	H_2_N-BDC	Water and AC	4	120		2.0 × 10^−9^ to 5.0 × 10^−14^ mol L^−1^	4.1 × 10^−14^ mol L^−1^	[12]
Immunosensing	Atrazine	Cu-MOF	Antibody	Cu (OAc)_2_·H_2_O and (TEOS)	(H_3_BTC)	Water ethanol and NaOH	2	RT	water	0.01 nM–1 μM	0.01 nM	[190]
electrochemical	Antibiotics (CAP and OTC)	UiO-66-NH_2_ and UiO-66	Aptamer	ZrCl_4_	H_2_N-H_2_BDC and H_2_BDC	DMF and AC	8	120	milk	0.0001–50 nM	CAP:33 fM OTC:48 fM	[205]
	Antibiotics (KANA CAP)	UiO-66-NH_2_	Aptamer	ZrCl_4_	H_2_N-H_2_BDC and H_2_BDC	DMF and AC	8	120	milk		KANA:0.16 pM CAP: 0.19 pM	[205]
electrochemical	kanamycin and neomycin	MIL-53(Fe)	Aptamer	FeCl_3_·6H_2_O	H_2_BDC	DMF	65	120	Milk and honey	1.0 × 10^−10^–1.0 × 10^−6^ M	1.7 × 10^−11^ M	[206]
	patulin (PAT)	UiO-66-NH_2_	Aptamer	ZrCl_4_	BDC-NH2	DMF	8	120	Apple Juice	5 × 10−8^−5^ × 10^−1^ μg mL^−1^	1.46 × 10^−8^ μg mL^−1^	[207]
Fluorescent	OTA	HKUST-1	Aptamer	Cu(NO_3_)_2_·3H_2_O	H_3_BTC	Water and ethanol	12	120	corn	5.0–160.0 ng/mL	2.57 ng/mL	[199]
	OTA	MOF-74	Aptamer	Cu(NO_3_)_2_·3H_2_O cadmium acetate dihydrate	DHTA	DMF	125	20	Red wine	0.05–100 ng mL^−1^	10 pg mL^−1^	[11]
Colorimetric	chloramphenicol	Fe-MIL-88	Aptamer	FeCl_3_·6H_2_O	terephthalic acid	DMF and AC	4	120	milk	0.1 pM–1000 pM	0.03 pM	[208]
Electrochemical	acetamiprid	Au-Cu-MOF	Aptamer	CuCl_2_	Trimesic acid (TMA)	Water and NaOH	RT	12	tea	0.1 pM to 10.0 nM	2.9 fM	[209]
Electrochemical	Tobramycin (TOB)	Ce/Cu-MOF	Aptamer	Ce(NO_3_)_3_·6H_2_O and Cu(NO_3_)_2_·3H_2_O	H_3_BTC	Ethanol and water	RT	24	Milk and human serum	0.01 pg mL^−1^–10 ng mL^−1^	2.0 fg mL^−1^	[210]
